# Current Treatment Strategies for Multiple Myeloma at First Relapse

**DOI:** 10.3390/jcm14051655

**Published:** 2025-02-28

**Authors:** Evangelos Mavrothalassitis, Konstantinos Triantafyllakis, Panagiotis Malandrakis, Maria Gavriatopoulou, Martina Kleber, Ioannis Ntanasis-Stathopoulos

**Affiliations:** 1 Department of Clinical Therapeutics, School of Medicine, National and Kapodistrian University of Athens, 11527 Athens, Greece; 2 Department of Internal Medicine, Clinic Hirslanden Zurich, 8032 Zurich, Switzerland; martina.kleber@usb.ch; 3 Faculty of Medicine, University of Basel, 4031 Basel, Switzerland

**Keywords:** multiple myeloma, relapsed/refractory, relapse, refractoriness, response, survival, immunotherapy, chimeric antigen receptor T-cells, belantamab mafodotin

## Abstract

Multiple myeloma (MM), the second most common hematologic cancer, remains an incurable malignancy, characterized by an initial response to therapy followed by successive relapses. The upfront treatment typically involves induction therapy, autologous stem cell transplantation for eligible patients, and long-term maintenance therapy. It is important to note that the anticipated duration of myeloma response diminishes with each subsequent relapse. Therefore, the first relapse represents a critical juncture in treatment, where refractoriness to key drug classes emerges as a significant challenge. Addressing the optimal management in this setting requires careful consideration of disease biology, prior therapies, and patient-specific factors to optimize outcomes. Cilta-cel, a chimeric antigen receptor T-cell construct, has emerged as the most promising therapeutic option at first relapse, resulting in long-term remissions with a significant treatment-free interval. However, availability and accessibility are not universal and treatment logistics are complex. Triplet regimens based on carfilzomib, pomalidomide or selinexor, remain the cornerstone of treatment at first relapse, whereas the optimal combination is based on refractoriness to prior drugs, especially anti-CD38 monoclonal antibodies and lenalidomide, and patient comorbidities. With the rapidly expanding therapeutic landscape, clinicians face increasing complexity in selecting the most appropriate regimens for individual patients. This review aims to guide clinicians through these evolving options by consolidating evidence-based strategies and highlighting emerging therapies, ensuring a personalized approach to managing first-relapse MM.

## 1. Introduction

### 1.1. Epidemiology

According to Global Cancer Observatory (GLOBOCAN) statistics, there were an estimated 160,000 cases of multiple myeloma (MM) globally in 2018, accounting for 0.9% of all cancer diagnoses. In 2018, an estimated 106,000 people globally died from MM, accounting for 1.1% of all cancer deaths. The current estimated incidence rate is 7.0/100,000. Among the risk factors are age above 65 years, male gender, African American descent and first degree relative with MM especially among men and African Americans [1].

### 1.2. General and Diagnosis

MM is an obstinate hematological malignancy, included in the broader spectrum of plasma cell dyscrasias, which originates from the post-germinal lymphoid B-cell lineage and develops after lineage commitment in the bone marrow of progenitor cells [2]. In the same spectrum of plasma cell dyscrasias that encompasses MM are also included a premalignant asymptomatic state called monoclonal gammopathy of unknown significance (MGUS), an intermediate state between MGUS and MM that is also asymptomatic, called smoldering myeloma (SMM), and lastly MM and plasma cell leukemia (PCL), diseases which are associated with end-organ damage and significant patient morbidity [3,4].

In order to diagnose a patient with MM the following criteria must be met [5]:

(1) ≥10% clonal bone marrow plasma cells or a biopsy proven plasmacytoma;
(2) Evidence of one or more multiple myeloma defining events (MDE):
  - CRAB (hypercalcemia, renal failure, anemia, or lytic bone lesions) attributable to the plasma cell disorder;
  - Bone marrow clonal plasmacytosis ≥60%;
  - Serum involved/uninvolved free light chain (FLC) ratio ≥100 (provided involved FLC is ≥100 mg/L);
  - >1 focal lesion on magnetic resonance imaging greater than 5 mm.

### 1.3. First Line Treatment

Regarding the first line treatment approach of MM, usually after risk stratification based on the revised international staging system [6,7], a patient is deemed either eligible or ineligible for autologous stem cell transplantation (ASCT). For those who are eligible for ASCT, the standard frontline regimen includes a quadruplet regimen consisting of an anti-CD38 monoclonal antibody (daratumumab or isatuximab), a PI (bortezomib), an IMiD drug (lenalidomide or thalidomide), and dexamethasone followed by an early ASCT and maintenance therapy with lenalidomide with or without daratumumab. For patients with high risk prognostic features, a tandem ASCT and the addition of bortezomib in the maintenance regimen may also be considered [8]. In the ineligible for the ASCT subgroup, patients usually receive continuous treatment with the combination of an anti-CD38 monoclonal antibody (daratumumab or isatuximab), lenalidomide, and dexamethasone, with or without bortezomib, until disease progression, that may be de-escalated following initial response to treatment [9,10,11]. Although modern therapies may result in deep responses and long-term remissions in up to 10% of patients, especially for those who have sustained minimal residual disease (MRD) negativity [12,13,14,15,16], most patients will eventually relapse.

### 1.4. Factors to Consider upon First Relapse

Several factors must be taken into consideration in order to determine the optimal treatment approach in a patient experiencing first MM relapse. These include disease, and treatment- and patient-related parameters. 

Among the disease factors that affect the decision to treat, the most important are the presentation and aggressiveness of the relapse. A biochemical slow progression or asymptomatic disease can be monitored closely, while a manifestation of end organ damage, such as renal failure or disease progression secondary to plasma cell leukemia or invasion to the central nervous system, warrant an immediate initiation of treatment [17,18,19].

Patient related factors that should be considered in order to select a treatment also include the functional age and medical comorbidities [20]. An assessment of functional status using a comprehensive assessment tool, such as a frailty score, which includes a patient’s ability to perform instrumental activities of daily living, and Charlson comorbidity index, is an effective way to screen for the ability to tolerate a particular regimen [21].

Arguably the most important factor in the treatment of first relapse is the exposure to prior treatment related factors. These include the regimen used for induction, the quality and durability of the elicited response, past and residual toxicities, whether the patient underwent ASCT or not according to ASCT eligibility status, and the treatment on which the disease progressed [22]. 

A cornerstone drug for the first line treatment of MM is lenalidomide and daratumumab/isatuximab, especially considering the fact that these agents are also administered as maintenance therapy. Thus, it is important to determine if the patient is sensitive or refractory to lenalidomide and an anti-CD38 monoclonal antibody when it comes to choosing the next regimen. Refractoriness to a drug is defined as no response to primary therapy or progression within 60 days following the last dose [23]. 

Taking all the above into consideration, the aim of this review is to assess and critically discuss the current and developing treatment strategies for MM at the time of first relapse, presented according to the refractoriness status to prior anti-myeloma drugs.

## 2. Materials and Methods

### 2.1. Lenalidomide Refractory Disease

Lenalidomide refractoriness has become increasingly common in the setting of MM at first relapse, largely due to its established use as maintenance therapy following ASCT or initial induction therapy [8]. Real-world studies have shown that the outcomes of lenalidomide refractory patients are far from optimal [24,25,26]. Various treatment options can be used for lenalidomide refractory patients, though they differ in terms of strength of supporting evidence. A bibliographic review identified four anti-CD38 antibody-based regimens: three daratumumab-based regimens in daratumumab, carfilzomib, dexamethasone (DKd), daratumumab, bortezomib, dexamethasone (DVd), daratumumab, pomalidomide, dexamethasone (DPd), and one isatuximab triplet in isatuximab, carfilzomib, dexamethasone (IsaKd). Other options include an IMiD-based triplet, pomalidomide, bortezomib, and dexamethasone (PVd), or a CAR T-cell therapy or belantamab mafodotin-based triplets (BPd or BVd), which have proven efficacy in clinical trials but are still pending regulatory approval for clinical use. All of the proposed regimens have proven their superiority over previous therapeutic options in phase 3 trials that include patients with one to three prior lines of therapy, a subset of which is lenalidomide refractory. Two more therapeutic regimens can also be considered in this setting: carfilzomib in combination with dexamethasone (Kd), and Selinexor plus bortezomib and dexamethasone (SelinexorVd). The key trials are summarized in Table 1 and Table 2. 

The anti-CD38 monoclonal antibody, daratumumab, combined with carfilzomib and dexamethasone (DKd), is one of the few regimens that, as proven in the CANDOR trial, has overall survival (OS) benefit against a previous standard of care of carfilzomib with dexamethasone (Kd). Median OS for DKd was 50.8 months vs. 43.6 months for Kd (hazard ratio (HR), 0.78; 95% Confidence Interval (CI), 0.60–1.03; *p* = 0.042). Trends toward improved OS also occurred in predefined subgroups, including patients’ refractory to lenalidomide (KdD, not reached vs. Kd, 38.2 months; HR, 0.69; 95% CI, 0.43–1.11) and there was also significant improvement in patients with high-risk cytogenetics (KdD, 34.3 months vs. Kd: 17.1 months; HR, 0.52; 95% CI, 0.29–0.94). These effects were reflected as expected in the higher MRD negativity achievement rates, MRD negative complete response (CR) rates and in the median PFS (mPFS), where the addition of daratumumab to Kd provided a statistically significant benefit [27].

In the CASTOR trial the addition of daratumumab to bortezomib and dexamethasone (DVd) showed an OS benefit (HR, 0.74; *p* = 0.0075) after 72.6 months of follow-up in a population consisting of 32.9% of patients refractory to IMiDs. Median OS was 49.6 months for DVd vs. 38.5 months for Vd. The daratumumab group, also achieved improved MRD negativity rates, CR rates and mPFS. Despite failure of the lenalidomide refractory subgroup to achieve statistically significant OS benefit (HR, 0.96; 95% CI, 0.67 to 1.38), it derived PFS benefit from treatment with DVd compared with Vd (mPFS, 7.8 months vs. 4.9 months; HR, 0.44; 95% CI, 0.28–0.68; *p* = 0.0002) [28,29]. However, in the modern therapeutic era, DVd results in only modest patient outcomes in the real world practice [30].

In the APOLLO trial the triplet of daratumumab plus pomalidomide and dexamethasone (DPd) was more efficacious than pomalidomide plus dexamethasone (Pd) in a population consisting of 80% of lenalidomide refractory individuals. Median PFS was 24.4 months for DPd vs. 17.6 months for Pd (HR, 0.73; *p* = 0.034). Median OS was 34.4 months (95% CI, 23.7–40.3) in the DPd group vs. 23.7 months (19.6–29.4) in the Pd group (HR, 0.82; 95% CI, 0.61–1.11; *p* = 0.20) [31].

Isatuximab is another monoclonal antibody that binds to a unique epitope on CD38 that is different from daratumumab and induces antitumor activity through multiple mechanisms, including the direct inhibition of CD38 ectozyme activity [32]. Isatuximab added to carfilzomib and dexamethasone (IsaKd) is another option for the treatment of relapsed disease that showed superior efficacy compared with carfilzomib and dexamethasone (Kd) in the IKEMA trial. After a median follow up of 44 months, the addition of Isa to Kd prolonged PFS both for the total study population and for lenalidomide refractory patients. For the total population, mPFS improved from 19.2 (95% CI, 15.8–25.0) months in the Kd group to 35.7 (95% CI, 25.8–44.0) months in the IsaKd group (HR, 0.58; 95.4% CI, 0.42–0.79). For the lenalidomide refractory patients at the time of the analysis, PFS was 45.62% for the IsaKd group, while it was 28.58% for the Kd group (HR, 0.586; 95% CI, 0.353–0.972). The MRD negativity rate was 33.5% vs. 15.4% (Odds-Ratio (OR), 2.78; 95% CI, 1.55–4.99), and the MRD negativity CR rate was 26.3% vs. 12.2%, with IsaKd vs. Kd. The OS data are not yet published [33]. Interestingly, a real world study has also shown the high efficacy and acceptable safety profile of IsaKd for patients with RRMM at first relapse [34].

OPTIMISMM is another important trial that provided data regarding the utility of pomalidomide in the setting of first relapse. In this trial, PVd significantly improved PFS when compared with Vd both in lenalidomide refractory patients (17.8 vs. 9.5 months; *p* = 0.0276), and in lenalidomide nonrefractory patients (22.0 vs. 12.0 months; *p* = 0.0491). Significant improvement was also observed in CR rate with 9.4% vs. 4.6% in lenalidomide refractory and 17% vs. 4% for PVd vs. Vd, respectively, in the lenalidomide non-refractory subgroup [35]. Although a trend towards improved median OS was observed in the PVd group it did not reach statistical significance (35.6 months in the PVd group vs. 31.6 months in the Vd group; *p* = 0.571). When adjusted for subsequent therapies, OS improved with PVd versus Vd (HR, 0.76; 95% CI, 0.619–0.931; *p* = 0.008) [36].

Newer drug classes have been tested in relapsed and refractory MM. Two recently published trials, DREAMM-7 and DREAMM-8, evaluated belantamab mafodotin, a B-cell maturation antigen (BCMA)-targeting antibody–drug conjugate [37]. In DREAMM-7, the combination of belantamab mafodotin, bortezomib, and dexamethasone (BVd) outperformed daratumumab, bortezomib, and dexamethasone (DVd) in terms of CR, MRD negativity, and mPFS. After a median of 28.2 months of follow-up, 35% of patients in the BVd group achieved a CR or better, compared to 17% in the DVd group. MRD-negative status combined with CR was seen in 25% of patients in the BVd group versus 10% in the DVd group. The median PFS was 36.6 months in the BVd group compared to 13.4 months in the DVd group (HR, 0.41; *p* < 0.001), with a substantial benefit in the lenalidomide refractory patients (HR, 0.37; 95% CI, 0.24–0.56). Overall survival at 18 months was 84% in the BVd group compared to 73% in the DVd group [38].

In the DREAMM-8 study, belantamab mafodotin was combined with pomalidomide and dexamethasone (BPd), and this regimen outperformed PVd in terms of PFS, 12-month OS, CR, and MRD negativity. After a median follow-up of 21.8 months, 12-month PFS was 71% with BPd compared to 51% with PVd (HR, 0.52; *p* < 0.001). BPd also resulted in higher CR rates (40% vs. 16%) and MRD-negativity status 24% vs. 5%. In the subgroup analysis, patients refractory to lenalidomide experienced a statistically significant benefit in PFS with a HR of 0.45 (95% CI, 0.31–0.65) [39].

Importantly, a more recent immunotherapy strategy, CAR T-cells, has also shown its value in the first relapse setting. In the CARTITUDE-4 trial, ciltacabtagene autoleucel (cilta-cel), a BCMA–directed CAR T-cell therapy was compared to the physician’s choice of treatment in lenalidomide refractory patients who had received one to three lines of treatment. The standard treatment group received the combination of either DPd or PVd. At a median follow-up of 15.9 months (range, 0.1 to 27.3), the mPFS was not reached in the cilta-cel group and was 11.8 months in the standard care group (HR, 0.26; 95% CI, 0.18 to 0.38; *p*< 0.001). The PFS rate at 12 months was 75.9% (95% CI, 69.4 to 81.1) in the cilta-cel group and 48.6% (95% CI, 41.5 to 55.3) in the standard-care group. More patients in the cilta-cel group than in the standard-care group had a CR or better (73.1% vs. 21.8%), for a risk ratio of 2.9 (95% CI, 2.3 to 3.7; *p* < 0.001) and absence of MRD (60.6% vs. 15.6%). Death from any cause was reported in 39 patients and 46 patients, respectively (HR, 0.78; 95% CI, 0.5 to 1.2) [40]. 

Selinexor, a potent, orally administered selective inhibitor of nuclear export that binds to the Cys528 residue in the cargo-binding pocket of XPO1, represents another promising option for lenalidomide refractory patients. In the BOSTON trial, Selinexor combined with bortezomib and dexamethasone (SelinexorVd) demonstrated prolonged PFS compared to bortezomib and dexamethasone (Vd) alone (mPFS: 13.93 vs. 9.46 months; HR, 0.70; 95% CI, 0.53–0.93; *p* = 0.0075). OS results were also favorable, although the median OS was not reached in the Selinexor arm after a 17.3-month follow-up (HR, 0.84; 95% CI, 0.57–1.23; *p* = 0.1852). Additionally, the overall response rate (ORR) was superior in the Selinexor arm (76.4% vs. 62.3%; *p* = 0.0012). While the trial did not specify the proportion of lenalidomide refractory patients, SelinexorVd remains a viable option due to its demonstrated superiority over Vd and the possibility of providing the patients with an IMiD-free interval, which could be important for subsequent pomalidomide efficacy [41]. In the real-world, SelinexorVd has shown inferior ORR compared to clinical trials; however, it was given also to bortezomib-refractory patients [42]. Dose modifications and supportive care measures are important to assure compliance to treatment [43].

Kd is another option in the physician’s arsenal, and was approved after the ENDEAVOR trial. Kd has shown efficacy, though it might be inferior in terms of survival benefit compared to other more modern combinations. Nonetheless, it is still a treatment that can be considered in specific situations taking into consideration patient frailty, significant comorbidities, or personal treatment preferences [44].

**Table 1:** Overview of major clinical trials including lenalidomide refractory RRMM patients at first relapse.

Study Name	Study Phase	Treatment Groups	Median Prior Lines of Therapy (Range)	Lenalidomide Refractory (%)	ORR/MRD Negativity		Median PFS (Months)	Median OS (Months)
CANDOR [27]	Phase 3	DKd	1 to 3	33%	84%	27.9%	28.4	50.8
		Kd			73%, *p* = 0.008	9.1%, OR = 4.22 (95% CI, 2.28–7.83	15.2 HR = 0.64 (95% CI, 0.49–0.83)	43.6, *p* = 0.042
CASTOR [28,29]	Phase 3	DVd	1 to 3	32.9%	85%	15.1%	16.7	49.6
		Vd			63%, *p* < 0.0001	1.6%, *p* < 0.0001	7.1 *p* < 0.0001	38.5
APOLLO [31]	Phase 3	DPd	≥1	79%	69%	-	24.4	34.4
		Pd		80%	46%, *p* < 0.0001	-	17.6, *p* = 0.034	23.7, *p* = 0.20
IKEMA [33]	Phase 3	IsaKd	1 to 3	31.8%	86.6%	33.5%	35.7	NR
		Kd		34.1%	83.7%	15.4%, OR 2.78, 95% CI: 1.55–4.99	19.2 (HR = 0.58, 95.4% CI: 0.42–0.79)	NR
OPTIMISMM [35]	Phase 3	PVd	1 to 3	57.1%	90.1%	-	20.73	35.6
		Vd			54.8%, *p* < 0.001	-	11.63, *p* < 0.0027 (only first relapse patients)	31.6, *p* = 0.571
DREAMM-7 [38]	Phase 3	BVd	≥1	34%	83%	38.7%	36.6	84% survival at 18 months
		DVd			71%	17.1%	13.4, *p* < 0.001	73% survival at 18 months
DREAMM-8 [39]	Phase 3	BPd	≥1	78%	77%	32.3%	NR, (12 months: 71%)	83% at 12 months
		PVd			72%	5.4%	12.7, (12 months: 51%, HR = 0.52 (95% CI, 0.37–0.73)	76% at 12 months
CARTITUDE-4 [40]	Phase 3	CAR T cells	1 to 3	100%	84.6%	60.6%	NR	84.1% at 12 months
		Standard of care			67.3%, HR = 2.2 (95% CI, 1.5 to 3.1; *p* < 0.001)	15.6%, HR: 2.2 (95% CI, 1.8 to 2.6; *p* < 0.001), OR: 8.7 (95% CI; 5.4–13.9)	11.8, *p* < 0.001	83.6% at 12 months
BOSTON [41]	Phase 3	Selinexor + Vd	1 to 3	NR	76.4%	5%	13.93	Not reached (median follow up 17.3 months)
		Vd			62.3%, *p* = 0.0012	4%	9.46 (HR = 0.70, 95% CI, 0.53–0.93, *p* = 0.0075)	25, (HR = 0.84, 95% CI, 0.57–1.23, *p* = 0·1852)
ENDEAVOR [44]	Phase 3	Kd	1 to 3	NR	-	-	-	47.6
		Vd			-	-	-	40.0, HR = 0.791 (95% CI, 0.648–0·964), *p* = 0.010

ORR: Overall response rate; MRD negativity: Minimal residual disease negativity; PFS: progression free survival; OS: overall survival; DKd: Daratumumab, Carflizomib and dexamethasone; Kd: Carflizomib and dexamethasone; OR: Odds ratio; HR: hazard ratio; DVd: Daratumumab, Bortezomib and dexamethasone; Vd: Bortezomib and dexamethasone; DPd: Daratumumab, Pomalidomide and dexamethasone; Pd: Pomalidomide and dexamethasone; IsaKd: Isatuximab, Carflizomib and dexamethasone; PVd: Pomalidomide, Bortezomib and dexamethasone; BVd: Belantamab mafodotin, Bortezomib and dexamethasone; BPd: Belantamab mafodotin, Pomalidomide and dexamethasone; NR: not reported.

**Table 2:** Overview of major clinical trials including RRMM patients at first relapse—results on lenalidomide refractory subgroups.

Study Name	Treatment Groups	Median Prior Lines of Therapy (Range)	Lenalidomide Refractory (%)	ORR/MRD Negativity		Median PFS (Months)	Median OS (Months)
CANDOR [27]	Dkd	1 to 3	33	90%	-	28.1	NR
	Kd			67%	-	11.1, HR = 0.46 (95% CI, 0.28–0.73)	38.2, HR = 0.69 (0.43–1.11)
CASTOR [28,29]	DVd	1 to 3	32.9%	-	-	7.8	28.9
	Vd			-	-	4.9, HR = 0.44 (95% CI, 0.28–0.68)	32.6, HR = 0.96 (95% CI, 0.67–1.38)
APOLLO [31]	DPd	≥1	79%	-	-	-	-
	Pd		80%	-	-	-	-
IKEMA [33]	IsaKd	1 to 3	-	-	29.8%	45% at 44 months	-
	Kd		-	-	11.9%	30% at 44 months, HR = 0.586 (95% CI, 0.353–0.972)	-
OPTIMISMM [35]	PVd	1 to 3	57.1%	85.9%	-	17.8	-
	Vd			50.8%, *p* < 0.001	-	9.5, *p* = 0.0276	-
DREAMM-7 [38]	BVd	≥1	34%	-	-	58.2% at 28.2 months	-
	DVd			-	-	26.4% at 28.2 months, HR = 0.37 (95% CI, 0.24–0.56)	-
DREAMM-8 [39]	BPd	≥1	78%	-	-	56.8% (12 month PFS)	-
	PVd			-	-	36.9% (12 month PFS), HR = 0.45 (95% CI, 0.31–0.65)	
CARTITUDE-4 [40]	CAR T cells	1 to 3	100%	84.6%	60.6%	NR	84.1% at 12 months
	Standard of care			67.3%, HR = 2.2 (95% CI, 1.5 to 3.1; *p* < 0.001)	15.6%, HR: 2.2 (95% CI, 1.8 to 2.6; *p* < 0.001)	11.8, *p* < 0.001	83.6% at 12 months

ORR: Overall response rate; MRD negativity: Minimal residual disease negativity; PFS: progression free survival; OS: overall survival; DKd: Daratumumab, Carflizomib and dexamethasone; Kd: Carflizomib and dexamethasone; OR: Odds ratio; HR: hazard ratio; DVd: Daratumumab, Bortezomib and dexamethasone; Vd: Bortezomib and dexamethasone; DPd: Daratumumab, Pomalidomide and dexamethasone; Pd: Pomalidomide and dexamethasone; IsaKd: Isatuximab, Carflizomib and dexamethasone; PVd: Pomalidomide, Bortezomib and dexamethasone; BVd: Belantamab mafodotin, Bortezomib and dexamethasone; BPd: Belantamab mafodotin, Pomalidomide and dexamethasone; NR: not reported.

### 2.2. Anti-CD38 Refractory Disease

The integration of daratumumab in the induction and consolidation/maintenance regimens for transplant-eligible patients [45], as well as its use in first-line therapy for transplant-ineligible patients [46,47], has led to an increasing number of cases of daratumumab-refractory disease at first relapse. This is a particularly challenging patient population, especially if there is double refractoriness to anti-CD38 treatment and lenalidomide [48]. This trend is expected to rise with the recent publication of the AURIGA trial demonstrating improved outcomes when daratumumab is added to lenalidomide as maintenance therapy [49]. This is further reinforced by the positive results of the IMROZ and the GMMG-HD7 trial that introduce isatuximab-based combinations in the upfront treatment for transplant-ineligible and -eligible patients with MM [50,51]. As these shifts in clinical practice are relatively recent, few clinical trials have specifically included patients at first relapse who are refractory to anti-CD38 monoclonal antibodies. The results of these trials are summarized in Table 3 and Table 4.

Currently, only two trials have specifically performed subgroup analysis on patients with one or more prior lines of therapy and resistance to daratumumab. The DREAMM-8 trial assessed the effectiveness of BPd compared to PVd by enrolling 302 patients, 24% of whom had disease refractory to anti-CD38 antibodies. The BPd group showed a significant improvement in several outcomes, including MRD negative status, CR rates, and PFS after a median follow-up of 21.8 months. Subgroup analysis showed that the improvement in PFS was also observed in patients refractory to anti-Cd38 antibodies, nevertheless, without reaching statistical significance (42.9% 12-month PFS vs. 30.6% 12-month PFS; (HR, 0.65; 95% CI, 0.36–1.18) [39]. 

The CARTITUDE-4 trial showcased the superiority of CAR-T cell therapy over standard of care treatment in the setting of first relapse. In this trial, patients refractory to daratumumab were included and made up 21.3% of the standard of care group and 23.1% of the cilta-cel group. As previously mentioned, PFS, MRD negativity, and CR rates were all in favor of the cilta-cel treatment in the total study population. A subgroup analysis on anti-CD38 refractory patients was performed for PFS. Cilta-cel showed a favorable effect in refractory to both an anti-CD38 antibody and an IMiD (HR, 0.26; 95% CI, 0.14–0.50), as well as in the triple-class refractory patients (refractory to an anti-CD38 antibody, an IMiD and a PI (HR, 0.15; 95% CI, 0.05–0.39) [40].

Although no other drug regimens have been tested systematically in the setting of first relapse in daratumumab-refractory populations in large phase 3 trials, it is suitable to consider other proven combinations as long as they do not include anti-CD38 antibodies. Thus, physicians should also keep BVd, Kd, IxaRd, PVd, SelinexorVd, and KRd in mind when evaluating treatment options for these patients.

**Table 3:** Overview of major clinical trials including daratumumab refractory RRMM patients at first relapse.

Study Name	Study Phase	Treatment Groups	Median Prior Lines of Therapy (Range)	Daratumumab Refractory	ORR/MRD Negativity		Median PFS (Months)	Median OS (Months)
DREAMM-8 [39]	Phase 3	BPd	≥1	24%	77%	32.3%	NR, (12 months: 71%)	83% at 12 months
		PVd			72%	5.4%	12.7, (12 months: 51%, HR = 0.52 (95% CI, 0.37–0.73)	76% at 12 months
CARTITUDE-4 [40]	Phase 3	CAR T cells	1 to 3	26.4%	84.6%	60.6%	NR	84.1% at 12 months
		Standard of care		22.7%	67.3%, HR = 2.2 (95% CI, 1.5 to 3.1; *p* < 0.001)	15.6%, HR: 2.2 (95% CI, 1.8 to 2.6; *p* < 0.001), OR: 8.7 (95% CI; 5.4–13.9)	11.8, *p* < 0.001	83.6% at 12 months
DREAMM-7 [38]	Phase 3	BVd	≥1	0%	83%	38.7%	36.6	84% survival at 18 months
		DVd			71%	17.1%	13.4, *p* < 0.001	73% survival at 18 months
ENDEAVOR [44]	Phase 3	Kd	1 to 3	0%	-	-	-	47.6
		Vd			-	-	-	40.0, HR = 0.791 (95% CI, 0.648–0.964), *p* = 0.010
OPTIMISMM [35]	Phase 3	PVd	1 to 3	0%	90.1%	-	20.73	NR
		Vd			54.8%, *p* < 0.001	-	11.63, *p* < 0.0027 (subgroup for first relapse patients)	NR
BOSTON [41]	Phase 3	Selinexor + Vd	1 to 3	0%	76.4%	5%	13.93	Not reached (median follow up 17.3 months)
		Vd			62.3%, *p* = 0.0012	4%	9.46 (HR = 0.70, 95% CI, 0.53–0.93, *p* = 0.0075)	25, (HR = 0.84, 95% CI, 0.57–1.23, *p* = 0.1852)
TOURMALINE-MM1 [52]	Phase 3	IxaRd	1 to 3	0%	78.3%	-	20.6	NR
		Rd			71.5%, *p* = 0.04	-	14.7 (HR = 0.74, 95% CI, 0.59–0.94, *p* = 0.01)	NR
ASPIRE [53,54]	Phase 3	KRd	1 to 3	0%	87.1%	-	26.3	48.3
		Rd			66.7%, *p*< 0.001	-	17.6, HR = 0.69 (95% CI, 0.57–0.83), *p* = 0.0001	40.4, HR, = 0.79; (95% CI, 0.67–0.95), *p* = 0.0045

ORR: Overall response rate; MRD negativity: Minimal residual disease negativity; PFS: progression free survival; OS: overall survival; OR: Odds ratio; HR: hazard ratio; PVd: Pomalidomide, Bortezomib and dexamethasone; BPd: Belantamab mafodotin, Pomalidomide and dexamethasone.

**Table 4:** Overview of major clinical trials including RRMM patients at first relapse—results on daratumumab refractory subgroups.

Study Name	Treatment Groups	Median Prior Lines of Therapy (Range)	Daratumumab Refractory	Median PFS (Months)
DREAMM-8 [39]	BPd	≥1	24%	42.9% (12 month PFS)
	PVd			30.6% (12 month PFS), HR = 0.65 (95% CI; 0.36–1.18)
CARTITUDE-4 [40]	CAR T cells	1 to 3	26.4%	-
	Standard of care		22.7%	HR = 0.26 (95% CI, 0.14–0.50)

ORR: Overall response rate; MRD negativity: Minimal residual disease negativity; PFS: progression free survival; OS: overall survival; OR: Odds ratio; HR: hazard ratio; PVd: Pomalidomide, Bortezomib and dexamethasone; BPd: Belantamab mafodotin, Pomalidomide and dexamethasone; CAR: chimeric antigen receptor.

### 2.3. Bortezomib Refractory Disease

Multiple trials, including CANDOR, APOLLO, Cilta-cel, DREAMM-8, and IKEMA, provide the foundation for treating patients who are refractory to bortezomib. Additionally, data from the ELOQUENT-2 and POLLUX trials reinforce the therapeutic efficacy of ERd and DRd, respectively, in this patient population. The data from these studies are presented in Table 5 and Table 6.

In the CANDOR trial, 29% of the population was refractory to bortezomib. As already mentioned [27], DKd was superior to Kd in terms of MRD negativity rates and PFS, but not OS. OS failed to reach statistical significance in patients refractory to proteasome inhibitors, with a median OS of 43.2 months vs. 30 months for the DKd and Kd subgroups, respectively (HR, 0.698; 95% CI, 0.448–1.087). Furthermore, a PFS benefit was observed with DKd vs. Kd in bortezomib and ixazomib refractory patients (HR, 0.65; 95% CI, 0.42–1.00) [27].

Another option for bortezomib refractory patients comes from the CARTITUDE-4 trial, where 26.4% of patients in the cilta-cel group and 22.7% in the standard of care group were refractory to bortezomib. As previously mentioned, the cilta-cell group resulted in favorable outcomes in terms of ORR, CR rate, MRD negativity rate and PFS, with OS data being immature. In the subgroup analysis, PFS data also favored the cilta-cel group in patients refractory to both proteasome inhibitors and IMiDs (HR, 0.24; 95% CI, 0.14–0.38) [40].

The triplet of daratumumab plus pomalidomide and dexamethasone (DPd) was more efficacious than pomalidomide plus dexamethasone (Pd) in terms of survival outcomes, although the ORR rates were rather similar in a population consisting of patients refractory to proteasome inhibitors (ORR 47% vs. 49% for DPd vs. Pd, respectively) or both lenalidomide and proteasome inhibitors (ORR, 42% vs. 42% for DPd vs. Pd, respectively) in the APOLLO trial. Median PFS was 24.4 months for DPd vs. 17.6 months for Pd (HR, 0.73; *p* = 0.034). Median OS was 34.4 months (95% CI, 23.7–40.3) in the DPd group versus 23.7 months (19.6–29.4) in the Pd group (HR, 0.82; 95% CI, 0.61–1.11; *p* = 0.20) [31].

In the DREAMM-8 study, BPd outperformed PVd regarding PFS, 12-month OS, CR, and MRD negativity. The study population consisted of 26% vs. 24% refractory to proteasome inhibitors (10% vs. 5% bortezomib refractory, for BPd and PVd, respectively). In the subgroup analysis for bortezomib exposed individuals, the PFS was significantly prolonged with BPd compared to PVd with a HR of 0.55 (95% CI; 0.38–0.78) [39].

Another trial that included patients refractory to bortezomib was the IKEMA trial. In this case, 31.3% of the patients enrolled in the IsaKd group and 35.8% in the Kd group were refractory to a proteasome inhibitor. Overall, the addition of isatuximab improved MRD negativity and CR rates and prolonged PFS, although sub-analyses on bortezomib refractory patients were not available [33]. 

In the POLLUX trial, DRd proved to be a therapeutically superior regimen compared to Rd in a population sensitive to lenalidomide but with a 20.6% refractoriness to bortezomib. After a median follow up of 79.7 months the median OS was 67.6 months (95% CI, 53.1–80.5) in the D-Rd arm versus 51.8 months (95% CI, 44.0–60.0) in the Rd arm. The HR for death was 0.73 in favor of the DRd group (95% CI, 0.58–0.91; *p* = 0.0044). For patients with one prior line of therapy, the median OS was 77.8 months with D-Rd compared with 57.7 months with Rd (HR, 0.75; 95% CI, 0.56–1.02). The HR was also in favor of the DRd group in bortezomib refractory patients, but there was not a statistically significant correlation (9HR, 0.85; 95% CI, 0.55–1.310). MRD negativity rates were significantly higher with D-Rd compared with Rd (33.2% vs. 6.7%; *p* < 0.0001). Median time to subsequent treatment was significantly increased with D-Rd versus Rd (69.3 vs. 23.1 months; HR, 0.40; 95% CI, 0.32–0.50; *p* < 0.0001) [55].

The ELOQUENT 2 trial evaluated the addition of elotuzumab to lenalidomide and dexamethasone (ERd) vs. Rd in population sensitive to lenalidomide but with a 22% resistance to bortezomib. ORR were 79% (95% CI, 74–83) in the elotuzumab group and 66% (95% CI, 60–71) in the control group (OR 1.9; 95% CI, 1.4–2.8; *p* < 0.001). Despite CR rates being worse in the Elotuzumab group (4% vs. 7%), the combined response (sCR+ CR+ VGPR) was better (33% vs. 28%). PFS and OS favored the addition of elotuzumab to Rd. The mPFS was 19.4 months (95% CI, 16.6–22.2) for the elotuzumab group versus 14.9 months (95% CI, 12.1–17.2) in the control group, with a hazard ratio of 0.70 (95% CI, 0.57–0.85; *p* < 0.001). Median OS was 8.7 months longer with the addition of elotuzumab to Rd (48.3 months with ERd and 39.6 months with Rd). Although there was a reduction of 18% in the risk of death with ERd versus Rd (HR, 0.82; 95.4% CI, 0.68–1.00; *p*  =  0.0408), the median OS was similar in patients with one prior line of therapy (43.7 vs. 44.1 months; HR, 1.00; 95% CI, 0.77–1.32) [56,57]. 

Ixazomib is an orally administered next generation proteasome inhibitor (PI). In a head-to-head comparison of ixazomib combined with lenalidomide and dexamethasone (IxaRd) versus Rd, the IxaRd regimen demonstrated a 5.9-month improvement in mPFS (20.6 months vs. 14.7 months, respectively, HR = 0.74, 95% CI, 0.59–0.94, *p* = 0.01). Similar improvements were observed in terms of ORR with more than 71% patients responding in both groups (*p* = 0.04). Although the study included only a small percentage of PI-refractory individuals (1% in the IxaRd group vs. 2% in the Rd group), IxaRd remains a viable treatment option for patients refractory to bortezomib [52,58]. Furthermore, two single-arm phase 2 studies have shown encouraging activity of the combination of ixazomib with daratumumab and dexamethasone in patients with RRMM and early disease relapse [59,60]. However, in the context of modern treatment regimens, ixazomib-based combinations may be preferred mainly for frail patients or those who cannot receive more active treatments.

In the ASPIRE trial, the addition of carfilzomib to lenalidomide and dexamethasone (KRd) significantly improved PFS, OS, and ORR compared to Rd. KRd demonstrated a 20.4% improvement in ORR (87.1% vs. 66.7%, *p* < 0.001), an 8.7-month increase in mPFS (26.3 months vs. 17.6 months; HR, 0.69; 95% CI, 0.57–0.83; *p* = 0.0001), and a 7.9-month increase in median OS (48.3 months vs. 40.4 months; HR, 0.79; 95% CI, 0.67–0.95; *p* = 0.0045). Real-world data have also confirmed the efficacy and safety of KRd, similar to clinical trial results [61,62]. While the ASPIRE trial excluded bortezomib refractory individuals, subsequent findings from the A.R.R.O.W and A.R.R.O.W.2 trials have demonstrated that carfilzomib can be effectively utilized in this population [53,54,63,64]. Kd is another option in the physician’s arsenal and was approved following the results of the ENDEAVOR trial [44].

### 2.4. Patients Sensitive to Lenalidomide, Bortezomib and Daratumumab

For patients who remain sensitive to lenalidomide, bortezomib, and daratumumab, treatment options are more varied and less restrictive. Several of the previously mentioned trials, such as ASPIRE, CASTOR, POLLUX, IKEMA, APOLLO, DREAMM-7, and CARTITUDE-4, included a substantial proportion of patients who were sensitive to all three drug classes. Therefore, combinations such as KRd, IsaKd, DVd, DRd, DPd, ERd, IsaKd, PVd, BPd, BVd, Kd and cilta-cel are all supported by strong clinical evidence and are reasonable options in this setting. It is up to the clinician’s judgement to decide which drugs to choose based on the aforementioned patient and disease-related factors. All the available options for these patients are summarized in Table 7. 

**Table 5:** Overview of major clinical trials including bortezomib refractory RRMM patients at first relapse.

Study Name	Study Phase	Treatment Groups	Median Prior Lines of Therapy (Range)	Bortezomib Refractory	ORR/MRD Negativity		Median PFS (Months)	Median OS (Months)
CANDOR [27]	Phase 3	DKd	1 to 3	29%	84%	27.9%	28.4	50.8
		Kd			73%, *p* = 0.008	9.1%, OR = 4.22 (95% CI, 2.28–7.83	15.2 HR = 0.64 (95% CI, 0.49–0.83)	43.6, *p*= 0.042
CARTITUDE-4 [40]	Phase 3	CAR T cells	1 to 3	26.4%	84.6%	60.6%	NR	84.1% at 12 months
		Standard of care		22.7%	67.3%, HR = 2.2 (95% CI, 1.5 to 3.1; *p* < 0.001)	15.6%, HR: 2.2 (95% CI, 1.8 to 2.6; *p* < 0.001), OR: 8.7 (95% CI; 5.4–13.9)	11.8, *p* < 0.001	83.6% at 12 months
APOLLO [31]	Phase 3	DPd	≥1	47% (PI)	69%	9%	12.4	34.4
		Pd		49%(PI)	46%, *p* < 0.0001	2%, *p* = 0.01	6.9, *p* = 0.0018	23.7, *p* = 0.20
DREAMM-8 [39]	Phase 3	BPd	≥1	10%	77%	32.3%	NR, (12 months: 71%)	83% at 12 months
		PVd		5%	72%	5.4%	12.7, (12 months: 51%, HR = 0.52 (95% CI, 0.37–0.73)	76% at 12 months
IKEMA [33]	Phase 3	IsaKd	1 to 3	31% (PI)	86.6%	33.5%	35.7	NR
		Kd		36% (PI)	83.7%	15.4%, OR = 2.78 (95% CI: 1.55–4.99)	19.2 (HR = 0.58, 95.4% CI: 0.42–0.79)	NR
ELOQUENT 2 [56,57]	Phase 3	ERd	1 to 3	22%	79%	-	19.4	48.3
		Rd			66%, *p* < 0.001	-	14.9, *p* < 0.001	39.6, HR = 0.82 (95.4% CI, 0.68–1.00), *p* = 0.0408
POLLUX [55]	Phase 3	DRd	1 to 3	19.9% (PI)	92.9%	33.2%	44.5	67.6
		Rd		16.3% (PI)	76.4%, *p* < 0.0001	6.7%, *p* < 0.0001	17.5, HR = 0.44 (95% CI, 0.35–0.55), *p* < 0.0001	51.8
TOURMALINE-MM1 [52]	Phase 3	IxaRd	1 to 3	1%	78.3%	-	20.6	NR
		Rd		2%	71.5%, *p* = 0.04	-	14.7 (HR = 0.74, 95% CI, 0.59–0.94, *p* = 0.01)	NR
ASPIRE [53,54]	Phase 3	KRd	1 to 3	0%	87.1%	-	26.3	48.3
		Rd			66.7%, *p* < 0.001	-	17.6, HR = 0.69 (95% CI, 0.57–0.83), *p* = 0.0001	40.4, HR, = 0.79; (95% CI, 0.67–0.95), *p* = 0.0045
ENDEAVOR [44]	Phase 3	Kd	1 to 3	0%	-	-	-	47.6
		Vd			-	-	-	40.0, HR = 0.791 (95% CI, 0.648–0·964), *p* = 0.010

ORR: Overall response rate; MRD negativity: Minimal residual disease negativity; PFS: progression free survival; OS: overall survival; DKd: Daratumumab, Carflizomib and dexamethasone; Kd: Carflizomib and dexamethasone; OR: Odds ratio; HR: hazard ratio; DPd: Daratumumab, Pomalidomide and dexamethasone; Pd: Pomalidomide and dexamethasone; IsaKd: Isatuximab, Carflizomib and dexamethasone; PVd: Pomalidomide, Bortezomib and dexamethasone; BPd: Belantamab mafodotin, Pomalidomide and dexamethasone; ERd: Elotuzumab, Lenalidomide and dexamethasone; Rd: Lenalidomide and dexamethasone; DRd: Daratumumab, Lenalidomide and dexamethasone.

**Table 6:** Overview of major clinical trials including RRMM patients at first relapse—results on proteasome inhibitor refractory subgroups.

Study Name	Study Phase	Treatment Groups	Median Prior Lines of Therapy (Range)	Bortezomib Refractory	Median PFS (Months)	Median OS (Months)
CANDOR [27]	Phase 3	DKd	1 to 3	29%	13.1 (bortezomib or ixazomib refractory)	43.2 (PI)
		Kd			8.7 (bortezomib or ixazomib refractory), HR = 0.65 (95% CI, 0.42–1.00)	30.0 (PI), HR = 0.70 (95% CI, 0.45–1.09)
CARTITUDE-4 [40]	Phase 3	CAR T cells	1 to 3	26.4%	-	-
		Standard of care		22.7%	PI + IMiD HR = 0.24 (95% CI, 0.14–0.38)	-
APOLLO [31]	Phase 3	DPd	≥1	47% (PI)	8.3	-
		Pd		49% (PI)	6.3, HR = 0.73 (95%CI, 0.49–1.08)	-
DREAMM-8 [39]	Phase 3	BPd	≥1	10%	-	-
		PVd		5%	-	-
IKEMA [33]	Phase 3	IsaKd	1 to 3	31% (PI)	-	-
		Kd		36% (PI)	-	-
ELOQUENT 2 [56,57]	Phase 3	ERd	1 to 3	22%	-	-
		Rd			-	-
POLLUX [55]	Phase 3	DRd	1 to 3	19.9% (PI)	34.3	47.7
		Rd		16.3% (PI)	11.3, HR = 0.40 (95% CI, 0.24–0.47), *p* = 0.0003	33.0, HR = 0.85 (95% CI, 0.55–1.31)

ORR: Overall response rate; MRD negativity: Minimal residual disease negativity; PFS: progression free survival; OS: overall survival; DKd: Daratumumab, Carflizomib and dexamethasone; Kd: Carflizomib and dexamethasone; OR: Odds ratio; HR: hazard ratio; DPd: Daratumumab, Pomalidomide and dexamethasone; Pd: Pomalidomide and dexamethasone; IsaKd: Isatuximab, Carflizomib and dexamethasone; PVd: Pomalidomide, Bortezomib and dexamethasone; BPd: Belantamab mafodotin, Pomalidomide and dexamethasone; ERd: Elotuzumab, Lenalidomide and dexamethasone; Rd: Lenalidomide and dexamethasone; DRd: Daratumumab, Lenalidomide and dexamethasone.

**Table 7:** Overview of major clinical trials on RRMM patients who are sensitive to bortezomib, lenalidomide and daratumumab, at first relapse.

Study Name	Study Phase	Treatment Groups	Median Prior Lines of Therapy (Range)	ORR/MRD Negativity		Median PFS (Months)	Median OS (Months)
CANDOR [27]	Phase 3	Dkd	1 to 3	84%	27.9%	28.4	50.8
		Kd		73%, *p* = 0.008	9.1%, OR = 4.22 (95% CI, 2.28–7.83	15.2 HR = 0.64 (95% CI, 0.49–0.83)	43.6, *p* = 0.042
CASTOR [28,29]	Phase 3	DVd	1 to 3	85%	15.1%	16.7	49.6
		Vd		63%, *p* < 0.0001	1.6%, *p* < 0.0001	7.1 *p* < 0.0001	38.5
APOLLO [31]	Phase 3	DPd	≥1	69%	-	24.4	34.4
		Pd		46%, *p* < 0.0001	-	17.6, *p* = 0.034	23.7, *p* = 0.20
IKEMA [33]	Phase 3	IsaKd	1 to 3	86.6%	33.5%	35.7	NR
		Kd		83.7%	15.4%, OR 2.78, 95% CI: 1.55–4.99	19.2 (HR = 0.58, 95.4% CI: 0.42–0.79)	NR
OPTIMISMM [35]	Phase 3	PVd	1 to 3	90.1%	-	20.73	NR
		Vd		54.8%, *p* < 0.001	-	11.63, *p* < 0.0027 (only first relapse patients)	NR
DREAMM-7 [38]	Phase 3	BVd	≥1	83%	38.7%	36.6	84% survival at 18 months
		DVd		71%	17.1%	13.4, *p* < 0.001	73% survival at 18 months
DREAMM-8 [39]	Phase 3	BPd	≥1	77%	32.3%	NR, (12 months: 71%)	83% at 12 months
		PVd		72%	5.4%	12.7, (12 months: 51%, HR = 0.52 (95% CI, 0.37–0.73)	76% at 12 months
CARTITUDE-4 [40]	Phase 3	CAR T cells	1 to 3	84.6%	60.6%	NR	84.1% at 12 months
		Standard of care		67.3%, HR = 2.2 (95% CI, 1.5 to 3.1; *p* < 0.001)	15.6%, HR: 2.2 (95% CI, 1.8 to 2.6; *p* < 0.001), OR: 8.7 (95% CI; 5.4–13.9)	11.8, *p* < 0.001	83.6% at 12 months
ASPIRE [53,54]	Phase 3	KRd	1 to 3	87.1	-	26.3	48.3
		Rd		66.7, *p* < 0.001	-	17.6, HR = 0.69 (95% CI, 0.57–0.83), *p* = 0.0001	40.4, HR = 0.79 (95% CI, 0.67–0.95), *p* = 0.0045
ELOQUENT 2 [56,57]	Phase 3	ERd	1 to 3	79%	-	19.4	48.3
		Rd		66%, *p* < 0.001	-	14.9, *p* < 0.001	39.6, HR = 0.82 (95.4% CI, 0.68–1.00), *p* = 0.0408
POLLUX [55]	Phase 3	DRd	1 to 3	92.9%	33.2%	44.5	67.6
		Rd		76.4%, *p* < 0.0001	6.7%, *p* < 0.0001	17.5, HR = 0.44 (95% CI, 0.35–0.55), *p* < 0.0001	51.8
TOURMALINE-MM1 [52]	Phase 3	IxaRd	1 to 3	78.3%	-	20.6	NR
		Rd		71.5%, *p* = 0.04	-	14.7 (HR = 0.74, 95% CI, 0.59–0.94, *p* = 0.01)	NR
BOSTON [41]	Phase 3	Selinexor + Vd	1 to 3	76.4%	5%	13.93	NR
		Vd		62.3%, *p* = 0.0012	4%	9.46 (HR = 0.70, 95% CI, 0.53–0.93, *p* = 0.0075)	25, (HR = 0.84, 95% CI, 0.57–1.23, *p* = 0·1852)
ENDEAVOR [44]	Phase 3	Kd	1 to 3	-	-	-	47.6
		Vd		-	-	-	40.0, HR = 0.791 (95% CI, 0.648–0·964), *p* = 0.010

ORR: Overall response rate; MRD negativity: Minimal residual disease negativity; PFS: progression free survival; OS: overall survival; DKd: Daratumumab, Carflizomib and dexamethasone; Kd: Carflizomib and dexamethasone; OR: Odds ratio; HR: hazard ratio; DVd: Daratumumab, Bortezomib and dexamethasone; Vd: Bortezomib and dexamethasone; DPd: Daratumumab, Pomalidomide and dexamethasone; Pd: Pomalidomide and dexamethasone; IsaKd: Isatuximab, Carflizomib and dexamethasone; PVd: Pomalidomide, Bortezomib and dexamethasone; BVd: Belantamab mafodotin, Bortezomib and dexamethasone; BPd: Belantamab mafodotin, Pomalidomide and dexamethasone; ERd: Elotuzumab, Lenalidomide and dexamethasone; Rd: Lenalidomide and dexamethasone; DRd: Daratumumab, Lenalidomide and dexamethasone; KRd: Carflizomib, lenalidomide and dexamethasone; NR: not reached.

### 2.5. Salvage ASCT

ASCT remains an acceptable therapeutic option for patients experiencing relapse after an initial ASCT. Both the ESMO guidelines and the American and European Associations for Bone and Marrow transplantation have proposed ASCT as a reasonable therapeutic alternative for patients who relapse after 18 months or more following their first ASCT, or after 36 months in those who receive lenalidomide maintenance therapy [8,65,66].

Two phase 3 trials have evaluated the role of ASCT in the setting of first relapse. The first, published in 2016, compared ASCT with weekly cyclophosphamide, a now outdated treatment in this setting. The ASCT group showed a PFS benefit of 19 months vs. 11 months in the cyclophosphamide group (HR 0.45, 95% CI 0.31–0.64, log-rank *p* < 0.0001). Similarly, the median OS was longer in the ASCT group at 67 months compared to 52 months in the cyclophosphamide group (HR 0.56; 95% CI, 0.35–0.90; *p* = 0.0169) [67]. 

The second phase 3 trial, published in 2020, compared salvage ASCT with Rd. While ASCT showed a trend towards better PFS and OS, it did not reach statistical significance. Median PFS was 20.7 months in the transplant and 18.8 months in the control arm (HR 0.87; 95% CI 0.65–1.16; *p*  =  0.34). Median OS was not reached in the transplant and was 62.7 months in the control arm (HR 0.81; 95% CI 0.52–1.28; *p*  =  0.37). It is important to note that 41 patients (29%) did not receive the assigned salvage ASCT mainly due to early disease progression, which might have hidden any survival benefit provided by the actual treatment. Multivariate landmark analyses from the time of ASCT showed superior PFS and OS (*p* = 0.0087 and *p* = 0.0057, respectively) in patients who received ASCT [68].

Additional data concerning the utility of ASCT at the time of first relapse come from older retrospective studies supporting salvage ASCT [69,70,71], but their validity in the current era of myeloma therapeutics is rather questionable. 

## 3. Discussion

This review highlights the significant advancements in the treatment of RRMM at first relapse, reflecting the growing diversity and innovation in therapeutic options. By 2020, the treatment paradigm had already shifted towards triplet regimens, as outlined in the ESMO guidelines, with the integration of drug combinations including anti-CD38 antibodies like daratumumab and isatuximab that have become the new standard of care in this setting [8].

For lenalidomide refractory patients, the 2020 ESMO guidelines recommended regimens such as SelinexorVd, DVd, PVd, DKd, IsaKd, and Kd. While these remain viable, significant advancements have reshaped the treatment landscape. This review highlights the inclusion of newer regimens, such as Cilta-cel, BPd, BVd, and DPd, offering additional therapeutic options. Although BPd and BVd have shown improvements over previous standards of care, BPd and BVd are still pending approval from the regulatory authorities and are not yet available in everyday clinical practice. Notably, several new agents have outperformed former standard treatments in terms of response rates and survival outcomes. DKd and IsaKd have demonstrated superiority over Kd, while BVd has outperformed DVd, and BPd has surpassed PVd in terms of PFS. Consequently, Kd, DVd, and PVd are now largely outperformed by modern combinations.

Bortezomib-refractory patients now have an expanded range of evidence-based options, including CAR T-cell therapies, DPd, IxaRd, KRd and the still pending-approval BPd, alongside previously established regimens such as DKd, DRd, IsaKd, ERd, and Kd. In contrast, treatment options for daratumumab-refractory patients remain more limited due to the reliance of many triplet regimens on anti-CD38 antibodies. In this challenging setting, cilta-cel stands out as the optimal evidence-based therapy. Interestingly, two recently published, retrospective, real-world studies showed that cilta-cel resulted in superior patient outcomes compared to ide-cel, although the risk for infections and delayed neurotoxicity was higher [72,73]. Furthermore, accessibility issues may also impact patient outcomes [74]. As previously mentioned, despite being an evidence-based option, BPd is still pending approval for clinical use.

Given the growing importance of CAR T-cell therapy in this setting, optimizing treatment sequencing is essential. The IMWG recommends avoiding high-dose alkylators and bendamustine in patients likely to undergo CAR T-cell therapy to preserve T-cell function. For those with a high disease burden, bridging therapy should be considered during the 4–6 weeks of CAR T-cell manufacturing, using short-duration agents with minimal risk of prolonged cytopenias or infections. Additionally, CAR T-cell therapy should be prioritized over BCMA-targeted antibody-drug conjugates due to its superior efficacy. Patients previously treated with PIs, IMiDs, monoclonal antibodies, corticosteroids, or their combinations can safely proceed with CAR T-cell therapy without concern [75].

The role of ASCT remains a critical consideration in the treatment algorithm, offering durable remissions in selected patients for those who have derived substantial PFS benefit following the first ASCT. However, it carries significant treatment-related mortality and morbidity, and no evidence-based superiority over modern standards of care has been conclusively demonstrated. 

Newer combinations like cilta-cel, BPd and BVd have shown promising results, with these combinations demonstrating superior PFS and improved response rates over standard-of-care treatments. In addition to cilta-cel, ide-cel—another BCMA-directed CAR T-cell product—has shown superior efficacy over standard-of-care treatments in later lines of therapy and could potentially be a valuable option in the first-relapse setting [76]. Furthermore, the BCL2 inhibitor, venetoclax, is also a suitable option for patients with RRMM at first relapse with t(11;14) either as monotherapy or in combination with standard anti-myeloma agents such as daratumumab or carfilzomib; however, there are no published data from large phase 3 studies [77]. The ongoing evaluation of these therapies in clinical trials will be essential in determining their future role in this context.

**Figure 1:**
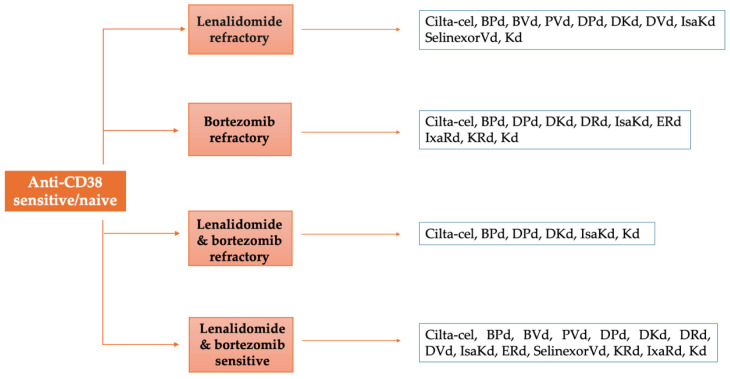
Proposed therapeutic algorithm for patients with multiple myeloma at first relapse who are anti-CD38 sensitive/naive based on disease refractoriness to prior treatments.

**Figure 2:**
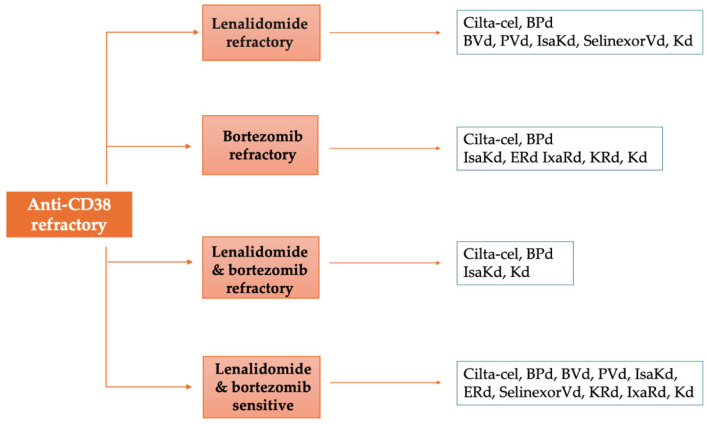
Proposed therapeutic algorithm for patients with multiple myeloma at first relapse who are anti-CD38 refractory based on disease refractoriness to prior treatments.

The growing complexity of therapeutic choices presents a challenge for clinicians, who have to determine the optimal drug combinations for individual patients (Figure 1 and Figure 2). This decision should be guided by factors such as refractoriness to prior treatments, disease characteristics, patient comorbidities, and practical factors such as drug availability, accessibility to hospital, and costs when formulating treatment strategies. Therefore, the clinician’s judgment for individualized patient treatment is of utmost importance in the context of limited comparative data from randomized controlled trials.

In addition, many emerging therapies are now being tested in the second line, as reflected by the numerous clinical trials registered in clinicaltrials.gov. These include bispecific antibodies including teclistamab, elranatamab, alnuctamab and talquetamab [78,79,80,81,82,83,84,85], next-generation CELMoD agents such as iberdomide and mezigdomide [86,87,88], newer CAR T cell products [89,90], and other agents like BCD-264, SG301 [91,92,93]. Also, new combinations including standard agents, such as selinexor with pomalidomide and dexamethasone or selinexor with carfilzomib and dexamethasone, are currently under evaluation to determine the optimum regimens that may improve patient outcomes [94]. All the ongoing trials concerning the first relapse setting registered on clinical trials.gov are summarized in Table 8.

## 4. Conclusions

The evolving treatment landscape for first-relapse MM underscores the need for an individualized approach to patient care. As innovative therapies such as CAR T cells, bispecific antibodies, and next-generation CELMoDs reshape the field, clinical trials remain essential in refining treatment strategies and expanding options for complex cases. By staying informed of ongoing advancements in the field, clinicians can ensure more personalized and effective care, ultimately improving outcomes for patients with RRMM.

## Figures and Tables

**Table 8:** Selected ongoing clinical trials including patients with RRMM at first relapse.

Title	NCT	Phase	Treatment	Estimated Enrollment
MagnetisMM-5: Study of Elranatamab (PF-06863135) Monotherapy and Elranatamab + Daratumumab Versus Daratumumab + Pomalidomide + Dexamethasone in Participants With Relapsed/Refractory Multiple Myeloma (MAGNETISMM-5)	NCT05020236	3	Elranatamab vs. D + Elranatamab vs. DPd	761
MagnetisMM-32: A Study to Learn About the Study Medicine Called Elranatamab in People With Multiple Myeloma (MM) That Has Come Back After Taking Other Treatments (Including Prior Treatment With an Anti-CD38 Antibody and Lenalidomide)	NCT06152575	3	Elranatanab vs. EPd, Kd or PVd	492
A Study of Teclistamab in Combination With Daratumumab Subcutaneously (SC) (Tec-Dara) Versus Daratumumab SC, Pomalidomide, and Dexamethasone (DPd) or Daratumumab SC, Bortezomib, and Dexamethasone (DVd) in Participants With Relapsed or Refractory Multiple Myeloma (MajesTEC-3)	NCT05083169	3	D + Tec vs. DPd or DVd	587
A Study Comparing Teclistamab Monotherapy Versus Pomalidomide, Bortezomib, Dexamethasone (PVd) or Carfilzomib, Dexamethasone (Kd) in Participants With Relapsed or Refractory Multiple Myeloma (MajesTEC-9)	NCT05572515	3	Tec vs. Kd or PVd	650
A Study Comparing Talquetamab in Combination With Daratumumab or in Combination With Daratumumab and Pomalidomide Versus Daratumumab in Combination With Pomalidomide and Dexamethasone in Participants With Multiple Myeloma That Returns After Treatment or is Resistant to Treatment (MonumenTAL-3)	NCT05455320	3	Tal + DP vs. DPd vs. Tal + D	810
A Study Comparing Talquetamab Plus Pomalidomide, Talquetamab Plus Teclistamab, and Elotuzumab, Pomalidomide, and Dexamethasone or Pomalidomide, Bortezomib, and Dexamethasone in Participants With Relapsed or Refractory Myeloma Who Have Received an Anti-CD38 Antibody and Lenalidomide (MonumenTAL-6)	NCT06208150	3	Tal + Tec vs. Tal + P vs. Epd or PVd	795
A Study to Evaluate Efficacy and Safety of Alnuctamab Compared to Standard of Care Regimens in Participants With Relapsed or Refractory Multiple Myeloma (RRMM) (ALUMMINATE)	NCT06232707	3	Alnuctamab vs. Standard of care regimens	0
A Trial to Learn How Well Linvoseltamab Works Compared to the Combination of Elotuzumab, Pomalidomide and Dexamethasone for Adult Participants With Relapsed/Refractory Multiple Myeloma (LINKER-MM3)	NCT05730036	3	Linvoseltamab vs. EloPd	380
A Phase III Study of Eque-cel in Subjects With Len-refractory RRMM (FUMANBA-03) (FUMANBA-03)	NCT06464991	3	Equecabtagene Autoleucel vs. DPd or PVd	240
A Study Comparing Anitocabtagene Autoleucel to Standard of Care Therapy in Participants With Relapsed/Refractory Multiple Myeloma (iMMagine-3)	NCT06413498	3	Anitocabtagene Autoleucel vs. Standard of care (PVd, DPd, DKd, Kd)	450
A Study to Evaluate Mezigdomide, Bortezomib and Dexamethasone (MEZIVd) Versus Pomalidomide, Bortezomib and Dexamethasone (PVd) in Participants With Relapsed or Refractory Multiple Myeloma (RRMM) (SUCCESSOR-1)	NCT05519085	3	MeziVd vs. PVd	810
Open-label Study Comparing Iberdomide, Daratumumab and Dexamethasone (IberDd) Versus Daratumumab, Bortezomib, and Dexamethasone (DVd) in Participants With Relapsed or Refractory Multiple Myeloma (RRMM) (EXCALIBER-RRMM)	NCT04975997	3	IberDd vs. DVd	864
A Study to Evaluate Mezigdomide in Combination With Carfilzomib and Dexamethasone (MeziKD) Versus Carfilzomib and Dexamethasone (Kd) in Participants With Relapsed or Refractory Multiple Myeloma (SUCCESSOR-2) (SUCCESSOR-2)	NCT05552976	3	MeziKd vs. Kd	525
Study Comparing Therapy for Advanced Relapsed/Refractory Multiple Myeloma With and Without Dexamethasone (FREEDOM)	NCT06561854	3	IsaKd or IsaPd vs. IsaKd or IsaPd with the dexamethasone discontinuation from the 3rd cycle	318
The Role of Ixazomib in Autologous Stem Cell Transplant in Relapsed Myeloma—Myeloma XII (ACCoRd)	NCT03562169	3	IxaTd INDUCTION plus conventional ASCT using melphalan, or augmented ASCT using melphalan with ixazomib	406
Study Comparing Continuous Versus Fixed Duration Therapy With Daratumumab, Lenalidomide, and Dexamethasone for Relapsed Multiple Myeloma (CONFIRM)	NCT03836014	3	DRd combination administered continuously until PD, versus a fixed duration of 24 months.	436
A Study of Combination of Selinexor, Pomalidomide, and Dexamethasone (SPd) Versus Elotuzumab, Pomalidomide, and Dexamethasone (EloPd) in Subject With Previously Treated Multiple Myeloma	NCT05028348	3	Selinexor + Pd vs. EPd	222
A Study of Evaluating the Safety and Efficacy of ATG-010, Bortezomib, and Dexamethasone (SVd) Versus Bortezomib and Dexamethasone (Vd) in Patients With Relapsed or Refractory Multiple Myeloma (RRMM) (BENCH)	NCT04939142	3	Selinexor + Vd vs. Vd	150
A Study of the Efficacy and Safety of Monotherapy With BCD-264 and Darzalex in Subjects With Relapsed and Refractory Multiple Myeloma (DARVIVA)	NCT06296121	3	BCD-264 vs. Darzalex	252
A Clinical Study Comparing SG301 Plus Pomalidomide and Dexamethasone to Placebo Plus Pomalidomide and Dexamethasone in Relapsed or Refractory Multiple Myeloma Patients	NCT06508983	3	SG301 + Pd vs. Placebo + Pd	360
A Study to Compare the Efficacy and Safety of BMS-986393 Versus Standard Regimens in Adult Participants With Relapsed or Refractory and Lenalidomide-refractory Multiple Myeloma (QUINTESSENTIAL-2)	NCT06615479	3	BMS-986393 vs. Standard regimens	440
To Evaluate the Efficacy and Safety of QL2109 and sc Daratumumab in Multiple Myeloma	NCT06742138	3	QL2109 + Pd vs. Pd + sc Daratumumab	284
TJ202, Lenalidomide and Dexamethasone vs. Lenalidomide and Dexamethasone in Subjects With Relapsed or Refractory Multiple Myeloma	NCT03952091	3	TJ202 + Rd vs. Rd	289
Allogeneic Stem Cell Transplantation vs. Conventional Therapy as Salvage Therapy for Relapsed/Progressive Patients With Multiple Myeloma After First-line Therapy (AlloRelapseMM)	NCT05675319	3	Allogeneic Stem Cell Transplantation vs. Currently approved triplet regimens at first relapse	482

SC: Subcutaneous; Tal: Talquetamab; D: Daratumumab; DPd: Daratumumab, Pomalidomide and dexamethasone; EPd: Elotuzumab, Pomalidomide and dexamethasone; Kd: Carflizomib and dexamethasone; PVd: Pomalidomide, Bortezomib and dexamethasone; Tec: Teclistamab; P: Pomalidomide; Pd: Pomalidomide and dexamethasone; DKd: Daratumumab, Carflizomib and dexamethasone; MeziVd: Mezigdomide (CC-92480), Bortezomib and dexamethasone; MeziKd: Mezigdomide, Carflizomib and dexamethasone; IberDd: Iberdomide, Daratumumab and dexamethasone; IsaKd: Isatuximab, Carflizomib and dexamethasone; IsaPd: Isatuximab, Pomalidomide and dexamethasone; DVd: Daratumumab, Bortezomib and dexamethasone; IxaTd: Izazomib, Thalidomide and dexamethasone; ASCT: Autologous stem cell transplantation; DRd: Daratumumab, lenalidomide and dexamethasone; Vd: Bortezomib and dexamethasone; Rd: Lenalidomide and dexamethasone.

## Data Availability

Further data are available upon reasonable request from the corresponding author. No new raw data were generated for this manuscript.

## References

[1] Padala S.A., Barsouk A., Barsouk A., Rawla P., Vakiti A., Kolhe R., Kota V., Ajebo G.H. (2021). Epidemiology, Staging, and Management of Multiple Myeloma. Med. Sci..

[2] Kazandjian D., Mailankody S., Korde N., Landgren O. (2014). Smoldering multiple myeloma: Pathophysiologic insights, novel diagnostics, clinical risk models, and treatment strategies. Clin. Adv. Hematol. Oncol..

[3] Landgren O. (2013). Monoclonal gammopathy of undetermined significance and smoldering multiple myeloma: Biological insights and early treatment strategies. Hematol. Am. Soc. Hematol. Educ. Program.

[4] Palumbo A., Anderson K. (2011). Multiple Myeloma. N. Engl. J. Med..

[5] Rajkumar S.V. (2022). Multiple myeloma: 2022 update on diagnosis, risk stratification, and management. Am. J. Hematol..

[6] D’Agostino M., Cairns D.A., Lahuerta J.J., Wester R., Bertsch U., Waage A., Zamagni E., Mateos M.V., Dall’Olio D., van de Donk N.W.C.J. (2022). Second Revision of the International Staging System (R2-ISS) for Overall Survival in Multiple Myeloma: A European Myeloma Network (EMN) Report Within the HARMONY Project. J. Clin. Oncol. Off. J. Am. Soc. Clin. Oncol..

[7] Palumbo A., Avet-Loiseau H., Oliva S., Lokhorst H.M., Goldschmidt H., Rosinol L., Richardson P., Caltagirone S., Lahuerta J.J., Facon T. (2015). Revised International Staging System for Multiple Myeloma: A Report from International Myeloma Working Group. J. Clin. Oncol. Off. J. Am. Soc. Clin. Oncol..

[8] Dimopoulos M.A., Moreau P., Terpos E., Mateos M.V., Zweegman S., Cook G., Delforge M., Hájek R., Schjesvold F., Cavo M. (2021). Multiple myeloma: EHA-ESMO Clinical Practice Guidelines for diagnosis, treatment and follow-up. Ann. Oncol..

[9] Kumar S.K., Mikhael J.R., Buadi F.K., Dingli D., Dispenzieri A., Fonseca R., Gertz M.A., Greipp P.R., Hayman S.R., Kyle R.A. (2009). Management of newly diagnosed symptomatic multiple myeloma: Updated Mayo Stratification of Myeloma and Risk-Adapted Therapy (mSMART) consensus guidelines. Mayo Clin. Proc..

[10] Mateos M.V., San Miguel J.F. (2017). Management of multiple myeloma in the newly diagnosed patient. Hematol. Am. Soc. Hematol. Educ. Program.

[11] Facon T., Kumar S.K., Plesner T., Orlowski R.Z., Moreau P., Bahlis N., Basu S., Nahi H., Hulin C., Quach H. (2021). Daratumumab, lenalidomide, and dexamethasone versus lenalidomide and dexamethasone alone in newly diagnosed multiple myeloma (MAIA): Overall survival results from a randomised, open-label, phase 3 trial. Lancet Oncol..

[12] Medina-Herrera A., Sarasquete M.E., Jiménez C., Puig N., García-Sanz R. (2023). Minimal Residual Disease in Multiple Myeloma: Past, Present, and Future. Cancers.

[13] Ravi P., Kumar S.K., Cerhan J.R., Maurer M.J., Dingli D., Ansell S.M., Rajkumar S.V. (2018). Defining cure in multiple myeloma: A comparative study of outcomes of young individuals with myeloma and curable hematologic malignancies. Blood Cancer J..

[14] Terpos E., Ntanasis-Stathopoulos I., Roussou M., Kanellias N., Fotiou D., Migkou M., Eleutherakis-Papaiakovou E., Gavriatopoulou M., Kastritis E., Dimopoulos M.A. (2020). Long PFS of more than 7 years is achieved in 9% of myeloma patients in the era of conventional chemotherapy and of first-generation novel anti-myeloma agents: A single-center experience over 20-year period. Ann. Hematol..

[15] Lehners N., Becker N., Benner A., Pritsch M., Löpprich M., Mai E.K., Hillengass J., Goldschmidt H., Raab M.S. (2018). Analysis of long-term survival in multiple myeloma after first-line autologous stem cell transplantation: Impact of clinical risk factors and sustained response. Cancer Med..

[16] Ntanasis-Stathopoulos I., Filippatos C., Ntanasis-Stathopoulos A., Malandrakis P., Kastritis E., Tsitsilonis O.E., Dimopoulos M.A., Terpos E., Gavriatopoulou M. (2025). Evaluating Minimal Residual Disease Negativity as a Surrogate Endpoint for Treatment Efficacy in Multiple Myeloma: A Meta-Analysis of Randomized Controlled Trials. Am. J. Hematol..

[17] Katodritou E., Dalampira D., Delimpasi S., Ntanasis-Stathopoulos I., Karaolidou F., Gkioka A.I., Labropoulou V., Spanoudakis E., Triantafyllou T., Kotsopoulou M. (2024). Central nervous system multiple myeloma: A real-world multi-institutional study of the Greek Myeloma Study Group. Am. J. Hematol..

[18] Dimopoulos M.A., Merlini G., Bridoux F., Leung N., Mikhael J., Harrison S.J., Kastritis E., Garderet L., Gozzetti A., van de Donk N.W.C.J. (2023). Management of multiple myeloma-related renal impairment: Recommendations from the International Myeloma Working Group. Lancet Oncol..

[19] Gavriatopoulou M., Musto P., Caers J., Merlini G., Kastritis E., van de Donk N., Gay F., Hegenbart U., Hajek R., Zweegman S. (2018). European myeloma network recommendations on diagnosis and management of patients with rare plasma cell dyscrasias. Leukemia.

[20] Ntanasis-Stathopoulos I., Gavriatopoulou M., Terpos E., Dimopoulos M.A. (2021). Real-World Treatment of Patients with Relapsed/Refractory Myeloma. Clin. Lymphoma Myeloma Leuk..

[21] Gahagan A., Maheshwari S., Rangarajan S., Ubersax C., Tucker A., Harmon C., Pasala M.S., Bal S., Godby K., Ravi G. (2024). Evaluating concordance between International Myeloma Working Group (IMWG) frailty score and simplified frailty scale among older adults with multiple myeloma. J. Geriatr. Oncol..

[22] Devarakonda S., Sharma N., Efebera Y. (2022). The first relapse in multiple myeloma: How to pick the next best thing. Hematol. Am. Soc. Hematol. Educ. Program.

[23] de Arriba de la Fuente F., Montes Gaisán C., de la Rubia Comos J. (2022). How to Manage Patients with Lenalidomide-Refractory Multiple Myeloma. Cancers.

[24] Dhakal B., Einsele H., Schecter J.M., Deraedt W., Lendvai N., Slaughter A., Lonardi C., Nair S., He J., Kharat A. (2024). Real-world treatment patterns and outcomes in relapsed/refractory multiple myeloma (1-3 prior lines): Flatiron database. Blood Adv..

[25] Hartley-Brown M.A., Weisel K., Bitetti J., Carter J.A., McNamara S., Purser M., Palumbo A., Richardson P.G. (2024). Multiple myeloma refractory to lenalidomide: A systematic literature review of trials and real-world evidence. Br. J. Haematol..

[26] Kastritis E., Ntanasis-Stathopoulos I., Theodorakakou F., Migkou M., Roussou M., Malandrakis P., Kanellias N., Eleutherakis-Papaiakovou E., Fotiou D., Spiliopoulou V. Characteristics and Outcomes of Patients with Relapsed/Refractory Multiple Myeloma After Exposure to Lenalidomide in First Line of Therapy: A Single Center Database Review in Greece-PubMed. https://pubmed.ncbi.nlm.nih.gov/38616479/.

[27] Usmani S.Z., Quach H., Mateos M.V., Landgren O., Leleu X., Siegel D., Weisel K., Shu X., Li C., Dimopoulos M. (2023). Final analysis of carfilzomib, dexamethasone, and daratumumab vs carfilzomib and dexamethasone in the CANDOR study. Blood Adv..

[28] Sonneveld P., Chanan-Khan A., Weisel K., Nooka A.K., Masszi T., Beksac M., Spicka I., Hungria V., Munder M., Mateos M.V. (2023). Overall Survival with Daratumumab, Bortezomib, and Dexamethasone in Previously Treated Multiple Myeloma (CASTOR): A Randomized, Open-Label, Phase III Trial. J. Clin. Oncol..

[29] Mateos M.V., Sonneveld P., Hungria V., Nooka A.K., Estell J.A., Barreto W., Corradini P., Min C.K., Medvedova E., Weisel K. (2020). Daratumumab, Bortezomib, and Dexamethasone Versus Bortezomib and Dexamethasone in Patients with Previously Treated Multiple Myeloma: Three-year Follow-up of CASTOR. Clin. Lymphoma Myeloma Leuk..

[30] Moore S., Cornic L., Crossman-Barnes C.J., Jose S., Khalaf Z., Yong K., Soutar M., Woods P. (2024). Real-world characteristics and outcomes of patients with multiple myeloma receiving second-line treatment in England. EJHaem.

[31] Dimopoulos M.A., Terpos E., Boccadoro M., Delimpasi S., Beksac M., Katodritou E., Moreau P., Baldini L., Symeonidis A., Bila J. (2023). Subcutaneous daratumumab plus pomalidomide and dexamethasone versus pomalidomide and dexamethasone in patients with relapsed or refractory multiple myeloma (APOLLO): Extended follow up of an open-label, randomised, multicentre, phase 3 trial. Lancet Haematol..

[32] Deckert J., Wetzel M.C., Bartle L.M., Skaletskaya A., Goldmacher V.S., Vallée F., Zhou-Liu Q., Ferrari P., Pouzieux S., Lahoute C. (2014). SAR650984, A Novel Humanized CD38-Targeting Antibody, Demonstrates Potent Antitumor Activity in Models of Multiple Myeloma and Other CD38+ Hematologic Malignancies. Clin. Cancer Res..

[33] Martin T., Dimopoulos M.A., Mikhael J., Yong K., Capra M., Facon T., Hajek R., Špička I., Baker R., Kim K. (2023). Isatuximab, carfilzomib, and dexamethasone in patients with relapsed multiple myeloma: Updated results from IKEMA, a randomized Phase 3 study. Blood Cancer J..

[34] De Novellis D., Derudas D., Vincelli D., Fontana R., Della Pepa R., Palmieri S., Accardi F., Rotondo F., Morelli E., Gigliotta E. (2025). Clinical Efficacy of Isatuximab Plus Carfilzomib and Dexamethasone in Relapsed/Refractory Multiple Myeloma Patients. Eur. J. Haematol..

[35] Dimopoulos M., Weisel K., Moreau P., Anderson LDJr White D., San-Miguel J., Sonneveld P., Engelhardt M., Jenner M., Corso A. (2021). Pomalidomide, bortezomib, and dexamethasone for multiple myeloma previously treated with lenalidomide (OPTIMISMM): Outcomes by prior treatment at first relapse. Leukemia.

[36] Richardson P., Beksaç M., Oriol A., Lindsay J., Schjesvold F., Galli M., Yağcı M., Larocca A., Weisel K., Yu X. (2025). Pomalidomide, Bortezomib, and Dexamethasone Versus Bortezomib and Dexamethasone in Relapsed or Refractory Multiple Myeloma: Final Survival and Subgroup Analyses from the Optimismm Trial. Eur. J. Haematol..

[37] Kleber M., Ntanasis-Stathopoulos I., Terpos E. (2021). BCMA in Multiple Myeloma-A Promising Key to Therapy. J. Clin. Med..

[38] Hungria V., Robak P., Hus M., Zherebtsova V., Ward C., Ho P.J., Ribas de Almeida A.C., Hajek R., Kim K., Grosicki S. (2024). Belantamab Mafodotin, Bortezomib, and Dexamethasone for Multiple Myeloma. N. Engl. J. Med..

[39] Dimopoulos M.A., Beksac M., Pour L., Delimpasi S., Vorobyev V., Quach H., Spicka I., Radocha J., Robak P., Kim K. (2024). Belantamab Mafodotin, Pomalidomide, and Dexamethasone in Multiple Myeloma. N. Engl. J. Med..

[40] San-Miguel J., Dhakal B., Yong K., Spencer A., Anguille S., Mateos M.V., Fernández de Larrea C., Martínez-López J., Moreau P., Touzeau C. (2023). Cilta-cel or Standard Care in Lenalidomide-Refractory Multiple Myeloma. N. Engl. J. Med..

[41] Grosicki S., Simonova M., Spicka I., Pour L., Kriachok I., Gavriatopoulou M., Pylypenko H., Auner H.W., Leleu X., Doronin V. (2020). Once-per-week selinexor, bortezomib, and dexamethasone versus twice-per-week bortezomib and dexamethasone in patients with multiple myeloma (BOSTON): A randomised, open-label, phase 3 trial. Lancet Lond. Engl..

[42] Kastritis E., Gavriatopoulou M., Solia E., Theodorakakou F., Spiliopoulou V., Malandrakis P., Ntanasis-Stathopoulos I., Migkou M., Kokkali N., Eleutherakis-Papaiakovou E. (2023). Real World Efficacy and Toxicity of Selinexor: Importance of Patient Characteristics, Dose Intensity and Post Progression Outcomes. Clin. Lymphoma Myeloma Leuk..

[43] Gavriatopoulou M., Chari A., Chen C., Bahlis N., Vogl D.T., Jakubowiak A., Dingli D., Cornell R.F., Hofmeister C.C., Siegel D. (2020). Integrated safety profile of selinexor in multiple myeloma: Experience from 437 patients enrolled in clinical trials. Leukemia.

[44] Dimopoulos M.A., Goldschmidt H., Niesvizky R., Joshua D., Chng W.J., Oriol A., Orlowski R.Z., Ludwig H., Facon T., Hajek R. (2017). Carfilzomib or bortezomib in relapsed or refractory multiple myeloma (ENDEAVOR): An interim overall survival analysis of an open-label, randomised, phase 3 trial. Lancet Oncol..

[45] Perrot A., Touzeau C., Lambert J., Hulin C., Caillot D., Karlin L., Arnulf B., Rey P., Garderet L., Macro M. (2025). Isatuximab, Carfilzomib, Lenalidomide, and Dexamethasone Induction in Newly Diagnosed Myeloma: Analysis of the MIDAS Trial. Blood.

[46] Usmani S.Z., Facon T., Hungria V., Bahlis N.J., Venner C.P., Braunstein M., Pour L., Martí J.M., Basu S., Cohen Y.C. (2025). Daratumumab plus bortezomib, lenalidomide and dexamethasone for transplant-ineligible or transplant-deferred newly diagnosed multiple myeloma: The randomized phase 3 CEPHEUS trial. Nat. Med..

[47] Askeland F.B., Haukås E., Slørdahl T.S., Klostergaard A., Alexandersen T., Lysén A., Abdollahi P., Nielsen L.K., Hermansen E., Schjesvold F. (2025). Isatuximab, bortezomib, lenalidomide, and limited dexamethasone in patients with transplant-ineligible multiple myeloma (REST): A multicentre, single-arm, phase 2 trial. Lancet Haematol..

[48] Ramasamy K., Vij R., Kuter D., Cella D., Durie B.G.M., Abonour R., Rifkin R.M., Ailawadhi S., Lee H.C., Cowan A.J. (2024). Real-World Treatment Patterns and Clinical Outcomes in Patients with Multiple Myeloma Previously Treated with Lenalidomide and an Anti-CD38 Monoclonal Antibody. Clin. Lymphoma Myeloma Leuk..

[49] Badros A., Foster L., Anderson LDJr Chaulagain C.P., Pettijohn E., Cowan A.J., Costello C., Larson S., Sborov D.W., Shain K.H. (2025). Daratumumab with lenalidomide as maintenance after transplant in newly diagnosed multiple myeloma: The AURIGA study. Blood.

[50] Mai E.K., Bertsch U., Pozek E., Fenk R., Besemer B., Hanoun C., Schroers R., von Metzler I., Hänel M., Mann C. (2024). Isatuximab, Lenalidomide, Bortezomib, and Dexamethasone Induction Therapy for Transplant-Eligible Newly Diagnosed Multiple Myeloma: Final Part 1 Analysis of the GMMG-HD7 Trial. J. Clin. Oncol..

[51] Facon T., Dimopoulos M.A., Leleu X.P., Beksac M., Pour L., Hájek R., Liu Z., Minarik J., Moreau P., Romejko-Jarosinska J. (2024). Isatuximab, Bortezomib, Lenalidomide, and Dexamethasone for Multiple Myeloma. N. Engl. J. Med..

[52] Moreau P., Masszi T., Grzasko N., Bahlis N.J., Hansson M., Pour L., Sandhu I., Ganly P., Baker B.W., Jackson S.R. (2016). Oral Ixazomib, Lenalidomide, and Dexamethasone for Multiple Myeloma. N. Engl. J. Med..

[53] Stewart A.K., Rajkumar S.V., Dimopoulos M.A., Masszi T., Špička I., Oriol A., Hájek R., Rosiñol L., Siegel D.S., Mihaylov G.G. (2015). Carfilzomib, Lenalidomide, and Dexamethasone for Relapsed Multiple Myeloma. N. Engl. J. Med..

[54] Siegel D.S., Dimopoulos M.A., Ludwig H., Facon T., Goldschmidt H., Jakubowiak A., San-Miguel J., Obreja M., Blaedel J., Stewart A.K. (2018). Improvement in Overall Survival with Carfilzomib, Lenalidomide, and Dexamethasone in Patients With Relapsed or Refractory Multiple Myeloma. J. Clin. Oncol..

[55] Dimopoulos M.A., Oriol A., Nahi H., San-Miguel J., Bahlis N.J., Usmani S.Z., Rabin N., Orlowski R.Z., Suzuki K., Plesner T. (2023). Overall Survival with Daratumumab, Lenalidomide, and Dexamethasone in Previously Treated Multiple Myeloma (POLLUX): A Randomized, Open-Label, Phase III Trial. J. Clin. Oncol..

[56] Lonial S., Dimopoulos M., Palumbo A., White D., Grosicki S., Spicka I., Walter-Croneck A., Moreau P., Mateos M.V., Magen H. (2015). Elotuzumab Therapy for Relapsed or Refractory Multiple Myeloma. N. Engl. J. Med..

[57] Dimopoulos M.A., Lonial S., White D., Moreau P., Weisel K., San-Miguel J., Shpilberg O., Grosicki S., Špička I., Walter-Croneck A. (2020). Elotuzumab, lenalidomide, and dexamethasone in RRMM: Final overall survival results from the phase 3 randomized ELOQUENT-2 study. Blood Cancer J..

[58] Terpos E., Ramasamy K., Maouche N., Minarik J., Ntanasis-Stathopoulos I., Katodritou E., Jenner M.W., Plonkova H., Gavriatopoulou M., Vallance G.D. (2020). Real-world effectiveness and safety of ixazomib-lenalidomide-dexamethasone in relapsed/refractory multiple myeloma. Ann. Hematol..

[59] Terpos E., Ntanasis-Stathopoulos I., Gavriatopoulou M., Katodritou E., Hatjiharissi E., Malandrakis P., Verrou E., Golfinopoulos S., Migkou M., Manousou K. (2024). Efficacy and safety of daratumumab with ixazomib and dexamethasone in lenalidomide-exposed patients after one prior line of therapy: Final results of the phase 2 study DARIA. Am. J. Hematol..

[60] Delimpasi S., Dimopoulos M.A., Straub J., Symeonidis A., Pour L., Hájek R., Touzeau C., Bhanderi V.K., Berdeja J.G., Pavlíček P. (2024). Ixazomib plus daratumumab and dexamethasone: Final analysis of a phase 2 study among patients with relapsed/refractory multiple myeloma. Am. J. Hematol..

[61] Garcia-Guiñon A., Charry P.A., Jimenez M., Sarra J., Delgado I., Segura de la Torre L., Santaliestra M., Garcia-Pintos M., Gonzalez Y., Senin A. (2025). Real-world evidence of Carfilzomib, Lenalidomide and Dexamethasone (KRD) Scheme in patients with relapsed/refractory multiple myeloma. Ann. Hematol..

[62] Lee J.H., Choi J., Min C.K., Park S.S., Jo J.C., Lee Y.J., Kim J.S., Eom H.S., Jung J., Moon J.H. (2024). Superior outcomes and high-risk features with carfilzomib, lenalidomide, and dexamethasone combination therapy for patients with relapsed and refractory multiple myeloma: Results of the multicenter KMMWP2201 study. Haematologica.

[63] Moreau P., Mateos M.V., Berenson J.R., Weisel K., Lazzaro A., Song K., Dimopoulos M.A., Huang M., Zahlten-Kumeli A., Stewart A.K. (2018). Once weekly versus twice weekly carfilzomib dosing in patients with relapsed and refractory multiple myeloma (A.R.R.O.W.): Interim analysis results of a randomised, phase 3 study. Lancet Oncol..

[64] Dimopoulos M.A., Coriu D., Delimpasi S., Špička I., Upchurch T., Fang B., Talpur R., Faber E., Beksac M., Leleu X. (2024). A.R.R.O.W.2: Once- vs twice-weekly carfilzomib, lenalidomide, and dexamethasone in relapsed/refractory multiple myeloma. Blood Adv..

[65] Giralt S., Garderet L., Durie B., Cook G., Gahrton G., Bruno B., Hari P., Lokhorst H., McCarthy P., Krishnan A. (2015). American Society of Blood and Marrow Transplantation, European Society of Blood and Marrow Transplantation, Blood and Marrow Transplant Clinical Trials Network, and International Myeloma Working Group Consensus Conference on Salvage Hematopoietic Cell Transplantation in Patients with Relapsed Multiple Myeloma. Biol. Blood Marrow Transpl. Transplant. J. Am. Soc. Blood Marrow Transplant..

[66] Miller K.C., Gertz M.A., Buadi F.K., Hayman S.R., Lacy M.Q., Dispenzieri A.A., Dingli D., Kapoor P., Gonsalves W.I., Kourelis T. (2019). The impact of re-induction prior to salvage autologous stem cell transplantation in multiple myeloma. Bone Marrow Transplant..

[67] Cook G., Ashcroft A.J., Cairns D.A., Williams C.D., Brown J.M., Cavenagh J.D., Snowden J.A., Parrish C., Yong K., Cavet J. (2016). The effect of salvage autologous stem-cell transplantation on overall survival in patients with relapsed multiple myeloma (final results from BSBMT/UKMF Myeloma X Relapse [Intensive]): A randomised, open-label, phase 3 trial. Lancet Haematol..

[68] Goldschmidt H., Baertsch M.A., Schlenzka J., Becker N., Habermehl C., Hielscher T., Raab M.S., Hillengass J., Sauer S., Müller-Tidow C. (2021). Salvage autologous transplant and lenalidomide maintenance vs. lenalidomide/dexamethasone for relapsed multiple myeloma: The randomized GMMG phase III trial ReLApsE. Leukemia.

[69] Dhakal B., D’Souza A., Kleman A., Chhabra S., Mohan M., Hari P. (2021). Salvage Second Transplantation in Relapsed Multiple Myeloma. Leukemia.

[70] Michaelis L.C., Saad A., Zhong X., Le-Rademacher J., Freytes C.O., Marks D.I., Lazarus H.M., Bird J.M., Holmberg L., Kamble R.T. (2013). Salvage Second Hematopoietic Cell Transplantation in Myeloma. Biol. Blood Marrow Transpl. Transplant. J. Am. Soc. Blood Marrow Transplant..

[71] Grövdal M., Nahi H., Gahrton G., Liwing J., Waage A., Abildgaard N., Pedersen P.T., Hammerstrøm J., Laaksonen A., Bazia P. (2015). Autologous stem cell transplantation versus novel drugs or conventional chemotherapy for patients with relapsed multiple myeloma after previous ASCT. Bone Marrow Transplant..

[72] Hansen D.K., Peres L.C., Dima D., Richards A., Shune L., Afrough A., Midha S., Dhakal B., Kocoglu M.H., Atrash S. (2025). Comparison of Standard-of-Care Idecabtagene Vicleucel and Ciltacabtagene Autoleucel in Relapsed/Refractory Multiple Myeloma. J. Clin. Oncol..

[73] Merz M., Albici A.M., von Tresckow B., Rathje K., Fenk R., Holderried T., Müller F., Tovar N., Oliver-Cáldes A., Vucinic V. (2025). Idecabtagene vicleucel or ciltacabtagene autoleucel for relapsed or refractory multiple myeloma: An international multicenter study. HemaSphere.

[74] Wesson W., Dima D., Suleman N., Saif M.S.I., Tabak C., Logan E., Davis J.A., McGann M., Furqan F., Mohan M. (2024). Timing of Toxicities and Non-Relapse Mortality Following CAR T Therapy in Myeloma. Transpl. Transplant. Cell Ther..

[75] Costa L.J., Banerjee R., Mian H., Weisel K., Bal S., Derman B.A., Htut M.M., Nagarajan C., Rodriguez C., Richter J. (2025). International myeloma working group immunotherapy committee recommendation on sequencing immunotherapy for treatment of multiple myeloma. Leukemia.

[76] Rodriguez-Otero P., Ailawadhi S., Arnulf B., Patel K., Cavo M., Nooka A.K., Manier S., Callander N., Costa L.J., Vij R. (2023). Ide-cel or Standard Regimens in Relapsed and Refractory Multiple Myeloma. New Engl. J. Med..

[77] Kleber M., Ntanasis-Stathopoulos I., Terpos E. (2023). The Role of t(11;14) in Tailoring Treatment Decisions in Multiple Myeloma. Cancers.

[78] Pfizer MagnetisMM-32: A Study to Learn About the Study Medicine Called Elranatamab in People with Multiple Myeloma (MM) That Has Come Back After Taking Other Treatments (Including Prior Treatment with an Anti-CD38 Antibody and Lenalidomide). https://clinicaltrials.gov/study/NCT06152575.

[79] Janssen Research Development, LLC. A Study Comparing Teclistamab Monotherapy Versus Pomalidomide, Bortezomib, Dexamethasone (PVd) or Carfilzomib, Dexamethasone (Kd) in Participants with Relapsed or Refractory Multiple Myeloma Who Have Received 1 to 3 Prior Lines of Therapy, Including an Anti-CD38 Monoclonal Antibody and Lenalidomide. https://clinicaltrials.gov/study/NCT05572515.

[80] Janssen Research Development, LLC. A Study Comparing Talquetamab in Combination with Pomalidomide (Tal-P), Talquetamab in Combination with Teclistamab (Tal-Tec), and Elotuzumab, Pomalidomide, and Dexamethasone (EPd) or Pomalidomide, Bortezomib, and Dexamethasone (PVd) in Participants with Relapsed or Refractory Myeloma Who Have Received 1 to 4 Prior Lines of Therapy Including an Anti-CD38 Antibody and Lenalidomide. https://clinicaltrials.gov/study/NCT06208150.

[81] Janssen Research Development, LLC. A Study Comparing Talquetamab in Combination with Daratumumab or in Combination with Daratumumab and Pomalidomide Versus Daratumumab in Combination with Pomalidomide and Dexamethasone in Participants with Multiple Myeloma That Returns After Treatment or is Resistant to Treatment (MonumenTAL-3). https://clinicaltrials.gov/study/NCT05455320.

[82] Pfizer MagnetisMM-5: Study of Elranatamab (PF-06863135) Monotherapy and Elranatamab + Daratumumab Versus Daratumumab + Pomalidomide + Dexamethasone in Participants with Relapsed/Refractory Multiple Myeloma (MAGNETISMM-5). https://clinicaltrials.gov/study/NCT05020236.

[83] Janssen Research Development, LLC. A Study of Teclistamab in Combination with Daratumumab Subcutaneously (SC) (Tec-Dara) Versus Daratumumab SC, Pomalidomide, and Dexamethasone (DPd) or Daratumumab SC, Bortezomib, and Dexamethasone (DVd) in Participants with Relapsed or Refractory Multiple Myeloma (MajesTEC-3). https://clinicaltrials.gov/study/NCT05083169.

[84] Celgene A Study to Evaluate Efficacy and Safety of Alnuctamab Compared to Standard of Care Regimens in Participants with Relapsed or Refractory Multiple Myeloma (RRMM) (ALUMMINATE). https://clinicaltrials.gov/study/NCT06232707.

[85] Regeneron Pharmaceuticals A Trial to Learn How Well Linvoseltamab Works Compared to the Combination of Elotuzumab, Pomalidomide and Dexamethasone for Adult Participants with Relapsed/Refractory Multiple Myeloma (LINKER-MM3). https://clinicaltrials.gov/study/NCT05730036.

[86] Celgene A Study to Evaluate Mezigdomide, Bortezomib and Dexamethasone (MEZIVd) Versus Pomalidomide, Bortezomib and Dexamethasone (PVd) in Participants with Relapsed or Refractory Multiple Myeloma (RRMM) (SUCCESSOR-1). https://clinicaltrials.gov/study/NCT05519085.

[87] Celgene Open-label Study Comparing Iberdomide, Daratumumab and Dexamethasone (IberDd) Versus Daratumumab, Bortezomib, and Dexamethasone (DVd) in Participants with Relapsed or Refractory Multiple Myeloma (RRMM) (EXCALIBER-RRMM). https://clinicaltrials.gov/study/NCT04975997.

[88] Bristol-Myers Squibb A Study to Evaluate Mezigdomide in Combination with Carfilzomib and Dexamethasone (MeziKD) Versus Carfilzomib and Dexamethasone (Kd) in Participants with Relapsed or Refractory Multiple Myeloma (SUCCESSOR-2) (SUCCESSOR-2). https://clinicaltrials.gov/study/NCT05552976.

[89] Kite, A Gilead Company A Study Comparing Anitocabtagene Autoleucel to Standard of Care Therapy in Participants with Relapsed/Refractory Multiple Myeloma (iMMagine-3). https://clinicaltrials.gov/study/NCT06413498.

[90] Nanjing IASO Biotechnology Co., Ltd. A Phase III Study of Eque-cel in Subjects with Len-refractory RRMM (FUMANBA-03) (FUMANBA-03). https://clinicaltrials.gov/study/NCT06464991.

[91] Biocad A Double-Blind, Randomized Clinical Study of the Efficacy and Safety of Monotherapy With BCD-264 and Darzalex® in Subjects with Relapsed and Refractory Multiple Myeloma. https://clinicaltrials.gov/study/NCT06296121.

[92] Hangzhou Sumgen Biotech Co., Ltd. A Phase 3 Randomized, Placebo-controlled, Double-blind, Multicenter Study Comparing SG301 in Combination with Pomalidomide and Dexamethasone Versus Placebo in Combination with Pomalidomide and Dexamethasone in Patients with Relapsed or Refractory Multiple Myeloma. https://clinicaltrials.gov/study/NCT06508983.

[93] Juno Therapeutics, Inc., a Bristol-Myers Squibb Company A Phase 3, Randomized, Open-Label, Multicenter Study to Compare the Efficacy and Safety of BMS-986393, a GPRC5D-directed CAR-T Cell Therapy, Versus Standard Regimens in Adult Participants with Relapsed or Refractory and Lenalidomide-refractory Multiple Myeloma. https://clinicaltrials.gov/study/NCT06615479.

[94] Stichting European Myeloma Network A Phase 3 Randomized, Open-label Trial of Selinexor, Pomalidomide, and Dexamethasone (SPd) Versus Elotuzumab, Pomalidomide, and Dexamethasone (EloPd) in Patients with Relapsed or Refractory Multiple Myeloma (RRMM). https://clinicaltrials.gov/study/NCT05028348.

